# *Porphyromonas gingivalis* Components/Secretions Synergistically Enhance Pneumonia Caused by *Streptococcus pneumoniae* in Mice

**DOI:** 10.3390/ijms222312704

**Published:** 2021-11-24

**Authors:** Teppei Okabe, Yosuke Kamiya, Takeshi Kikuchi, Hisashi Goto, Masayuki Umemura, Yuki Suzuki, Yoshihiko Sugita, Yoshikazu Naiki, Yoshiaki Hasegawa, Jun-ichiro Hayashi, Shotaro Kawamura, Noritaka Sawada, Yuhei Takayanagi, Takeki Fujimura, Naoya Higuchi, Akio Mitani

**Affiliations:** 1 Department of Periodontology, School of Dentistry, Aichi Gakuin University, 2-11 Suemori-dori, Chikusa-ku, Nagoya 464-8651, Japan; ag183d03@dpc.agu.ac.jp (T.O.); tkikuchi@dpc.agu.ac.jp (T.K.); hisashi@dpc.agu.ac.jp (H.G.); ag193d11@dpc.agu.ac.jp (Y.S.); jun1row@dpc.agu.ac.jp (J.H.); ag213d08@dpc.agu.ac.jp (S.K.); nsawada@dpc.agu.ac.jp (N.S.); garound6@dpc.agu.ac.jp (Y.T.); takeki@dpc.agu.ac.jp (T.F.); minita@dpc.agu.ac.jp (A.M.); 2 Molecular Microbiology Group, Department of Tropical Infectious Diseases, Tropical Biosphere Research Center, University of the Ryukyus, 1 Senbaru, Nishihara-cho, Nakagami-gun, Nishihara 903-0213, Japan; umemura@comb.u-ryukyu.ac.jp; 3 Department of Oral Pathology and Forensic Odontology, School of Dentistry, Aichi Gakuin University, 1-100 Kusumoto-cho, Chikusa-ku, Nagoya 464-8650, Japan; yosshii@dpc.agu.ac.jp; 4 Department of Microbiology, School of Dentistry, Aichi Gakuin University, 1-100 Kusumoto-cho, Chikusa-ku, Nagoya 464-8650, Japan; naikiy@dpc.agu.ac.jp (Y.N.); yhase@dpc.agu.ac.jp (Y.H.); 5 Department of Endodontics, School of Dentistry, Aichi Gakuin University, 2-11 Suemori-dori, Chikusa-ku, Nagoya 464-8651, Japan; kinchan@dpc.agu.ac.jp

**Keywords:** *Porphyromonas gingivalis*, *Streptococcus pneumoniae*, pneumonia

## Abstract

*Streptococcus pneumoniae* is an important causative organism of respiratory tract infections. Although periodontal bacteria have been shown to influence respiratory infections such as aspiration pneumonia, the synergistic effect of *S. pneumoniae* and *Porphyromonas gingivalis*, a periodontopathic bacterium, on pneumococcal infections is unclear. To investigate whether *P. gingivalis* accelerates pneumococcal infections, we tested the effects of inoculating *P. gingivalis* culture supernatant (PgSup) into *S. pneumoniae*-infected mice. Mice were intratracheally injected with *S. pneumoniae* and PgSup to induce pneumonia, and lung histopathological sections and the absolute number and frequency of neutrophils and macrophages in the lung were analyzed. Proinflammatory cytokine/chemokine expression was examined by qPCR and ELISA. Inflammatory cell infiltration was observed in *S. pneumoniae*-infected mice and *S. pnemoniae* and PgSup mixed-infected mice, and mixed-infected mice showed more pronounced inflammation in lung. The ratios of monocytes/macrophages and neutrophils were not significantly different between the lungs of *S. pneumoniae*-infected mice and those of mixed-infected mice. PgSup synergistically increased TNF-α expression/production and IL-17 production compared with *S. pneumoniae* infection alone. We demonstrated that PgSup enhanced inflammation in pneumonia caused by *S. pneumoniae*, suggesting that virulence factors produced by *P. gingivalis* are involved in the exacerbation of respiratory tract infections such as aspiration pneumonia.

## 1. Introduction

Pneumonia is a globally common infection and a major cause of death, as highlighted by the impact of coronavirus disease 2019 (COVID-19) [1,2]. *Streptococcus pneumoniae*, a Gram-positive aerobic diplococci, is the main cause of bacterial pneumonia [3]. Aspiration pneumonia (AP) is defined as inflammation of the lung tissue, caused by bacterial infection resulting from the entry of foreign materials into the bronchial tree and lungs, usually from oral intake. The mortality and morbidity associated with AP is expected to increase over the next few decades, especially among the older population [4]. Most cases of AP are considered to be caused by a mixed infection of aerobic and anaerobic bacteria [5]. The oral cavity has long been suspected to be a source of the organisms responsible for AP. Bartlett and colleagues demonstrated the presence of oral anaerobic organisms in sputum by collecting samples directly from the trachea to avoid oral contamination [6]. The oral bacterial species implicated in causing pneumonia and lung abscesses are *Porphyromonas gingivalis*, *Aggregatibacter actinomycetemcomitans*, *Actinomyces israelii*, *Capnocytophaga species*, *Eikenella corrodens*, *Prevotella intermedia*, and *Streptococcus constellatus* [7]. These bacteria are closely associated with oral disease and oral hygiene. Systematic reviews of the relationship between pneumonia and oral health have provided evidence that oral care can reduce the exacerbation or incidence of respiratory illness in older people residing in nursing homes or patients in intensive care [8,9]. However, recent reports have questioned the quality of the evidence indicating that professional oral care reduces pneumonia mortality compared with regular care in nursing home residents, and so caution is therefore required in interpreting the results, as no conclusions have been reached regarding other outcomes effective in reducing pneumonia [10]. Regarding the relationship between *S. pneumoniae* and oral hygiene, Okuda and coworkers reported that oral lavage significantly decreased the percentage of *S. pneumoniae* bacteria detected in patients who underwent oral and maxillofacial surgery [11].

*S. pneumoniae* initially colonizes the nasopharynx and then invades the lower respiratory tract, causing pneumonia [12]. In the lungs, alveolar macrophages act as the first line of defense to eliminate pathogens, while also maintaining lung homeostasis by suppressing inflammation and promoting the regeneration of lung epithelial cells [13]. However, if elimination by alveolar macrophages is not sufficient, proinflammatory cytokines/chemokines such as tumor necrosis factor (TNF)-α produced by macrophages and interleukin (IL)-17 produced by γδ T cells at early stages of the immune response, are induced to promote neutrophil migration and eliminate pneumococci by phagocytosis [14,15].

Periodontitis is an infectious, chronic inflammatory disease induced by multiple periodontopathic bacteria [16]. Socransky and colleagues reported that three Gram-negative anaerobic bacteria, namely *P. gingivalis*, *Tanerella forsythia*, and *Treponema denticola*, are central to the onset and progression of periodontitis, and defined these three bacteria as the “red complex” [17]. Of these three species, *P. gingivalis* is the major pathogen associated with periodontitis. *P. gingivalis* has been reported to be more common in affected areas than in healthy areas in patients with periodontitis [18]. *P. gingivalis* possesses many virulence factors, such as lipopolysaccharide (LPS), gingipain, and fimbriae, and has a direct effect on periodontal tissue destruction [19]. *P. gingivalis* is thought to play an important role as a keystone pathogen that causes dysbiosis in oral biofilms. 

However, *P. gingivalis* is rarely the direct pathogenic cause of AP, and its impact on the development of AP has not been clarified. *P. gingivalis* is difficult to grow in an aerobic environment such as the lungs, and aspiration of small amounts of oral bacteria is dispelled by the defense mechanisms described above. Hence, rather than causing pneumonia through the growth of living *P. gingivalis*, it is possible that the bacterial components and products of the bacterium support infection with other bacteria such as *S. pneumoniae*—thereby establishing pneumonia. Therefore, we hypothesized that periodontal bacteria such as *P. gingivalis* have a synergistic effect on pneumococcal respiratory infections. In this study, the effects of the bacterial products of *P. gingivalis* on pneumococcal infection in mice were investigated using histological and immunological methods. The purpose of this study was to determine whether the periodontopathic bacterium *P. gingivalis* has a synergistic effect on pneumococcal pneumonia. 

## 2. Results

### 2.1. P. gingivalis Culture Supernatant (PgSup) Enhanced the Inflammation of S. pneumoniae (Sp) Infection, as Observed by Lung Histology and Flow Cytometric Analysis

**Figure 1:**
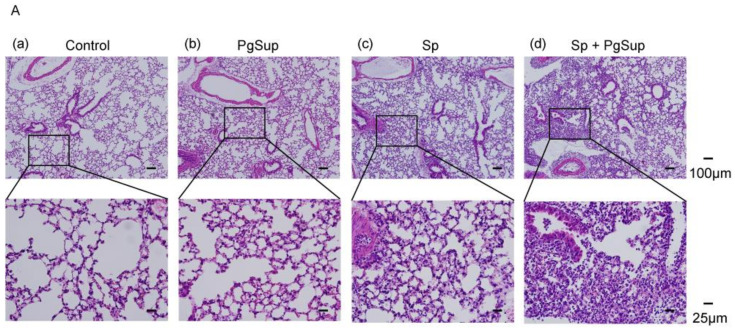

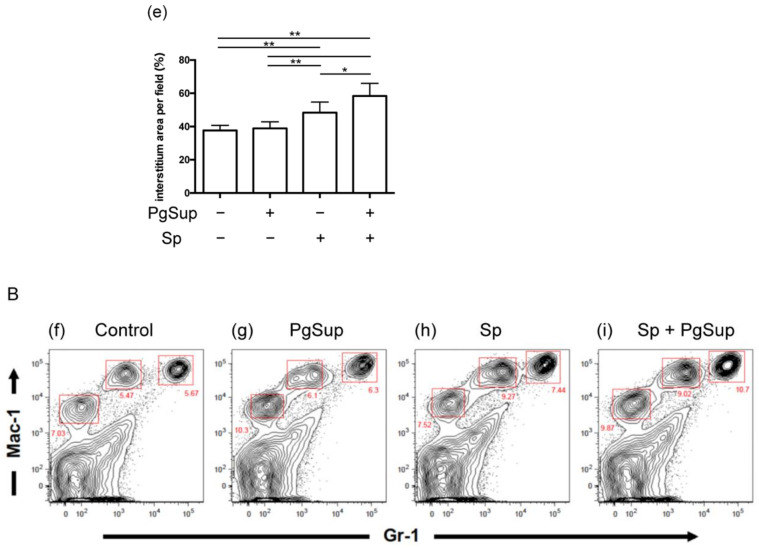
Histopathological analysis and cellular composition during the inflammatory cell infiltration of Sp-infected mice with and without PgSup. (**A**) The mice were euthanized 24 h after inoculation and lung collection was performed. (**a**–**d**) HE-stained histological section images are shown: (**a**) sTSB administration, (**b**) PgSup administration, (**c**) Sp administration, (**d**) Sp and PgSup mixed administration. The boxed regions are magnified in the lower images. Original magnification: ×10 (upper images), ×40 (lower images). (**e**) The percentage of interstitium in areas of the HE-stained histological sections. “+” indicates the presence of PgSup or Sp, and “−” indicates the absence of PgSup or Sp. Each group was analyzed by one-way ANOVA, and data are presented as the mean ± SD. Significant differences were analyzed using the Kruskal–Wallis test (* *p* < 0.05, ** *p* < 0.01). (**B**) Mice were sacrificed 24h after administration with (**f**) sTSB, (**g**) PgSup, (**h**) Sp, or (**i**) Sp-PgSup mixed. The cells were prepared from the lung and stained with Mac-1 and Gr-1 mAbs for flow cytometric analysis. The dots surrounded by a square represent neutrophils and monocytes/macrophages. Representative results from three separate experiments are shown in each panel.

Due to the aerobic environment in the lung, infection with anaerobic *P. gingivalis*, even at the maximum number possible in the oral cavity, may be difficult. In preliminary tests of *P. gingivalis* bacteria performed to investigate the possibility of any synergistic effects on lung Sp infection, no changes were observed following stimulation with *P. gingivalis* (data not shown). Thus, instead, we used the bacterial components/secretions (culture supernatant) of *P. gingivalis* rather than the bacteria itself in subsequent experiments. First, histopathology was evaluated by HE staining. In the control mice and the PgSup-only mice, no inflammatory findings such as interstitial thickening or inflammatory cell infiltration were observed (Figure 1A a,b), whereas in the Sp-infected mice and the Sp and PgSup mixed-infected mice, interstitial thickening and inflammatory cell infiltration were observed (Figure 1A c,d). In addition, we used ImageJ to quantitatively measure the percentage of lung interstitium in the area of HE-stained histological sections. Sp and PgSup mixed-infected mice showed a significant increase in the percentage of interstitium in the field compared to the Sp-infected mice (Figure 1A e; *p* < 0.05). Although inflammation was induced by Sp infection alone, it was observed that the inflammation was exacerbated by the presence of PgSup.

Next, to further compare cellular composition during the inflammatory cell infiltration of all the analyzed mouse groups, flow cytometric analysis of monocyte and granulocyte lineage markers was conducted on pulmonary infiltrated cells after administration. The ratios of CD11bhigh Gr-1high neutrophils (right square) were slightly higher in the lungs of the Sp and PgSup mixed-administrated mice than those in the Sp-administrated mice (Figure 1B h,i and Table 1). The ratios of CD11blow/dim Gr-1dim monocytes/macrophages (left and middle square) were the same between the Sp and PgSup mixed-administrated mice and the Sp-administrated mice. In addition, the ratio of macrophages to granulocytes in the administrated lungs of PgSup alone was nearly identical to that in the control mice (Figure 1B f,g and Table 1). 

### 2.2. PgSup Increased the Number of S. pneumoniae in the Lung

**Figure 2:**
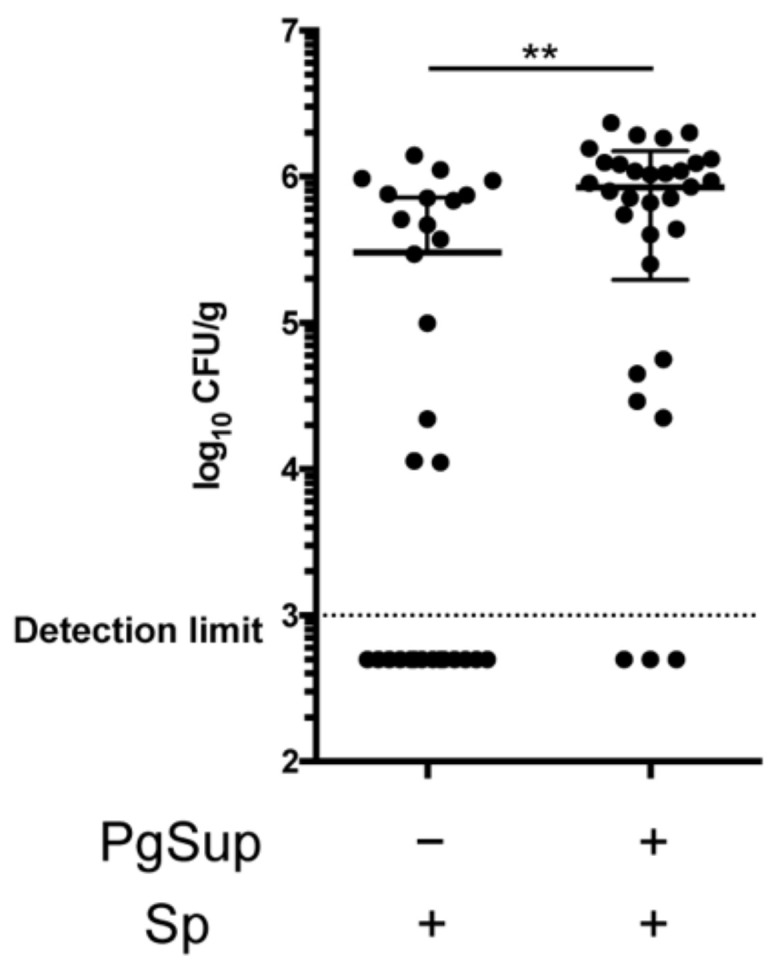
The number of *S. pneumoniae* in the lungs after 24 h infection following Sp administration and Sp and PgSup administration. “+” indicates the presence of PgSup or Sp, and “−” indicates the absence of PgSup or Sp. Each point in the graph represents an individual mouse. Samples below the detection limit (>8000 CFU/g) were standardized to 4000 CFU/g. Two groups were analyzed by an unpaired t-test, and data are presented as the mean ± SD (** *p* < 0.01).

Since we were able to confirm that PgSup exacerbated the inflammation caused by Sp infection, we then examined the number of *S. pneumoniae* in the lungs 24 h after infection. The CFU per gram of lung tissue in Sp and PgSup mixed-infected mice (*n* = 31) were significantly increased compared with those in Sp-infected mice (*n* = 30; *p* < 0.01; Figure 2). Mice with CFU values below the detection limit were observed in both groups, but the number of mice below the detection limit was lower in the presence of PgSup.

### 2.3. PgSup Enhanced Pro-Inflammatory Cytokine and Chemokine Gene Expression in the Lungs of Sp-Infected Mice

**Figure 3:**
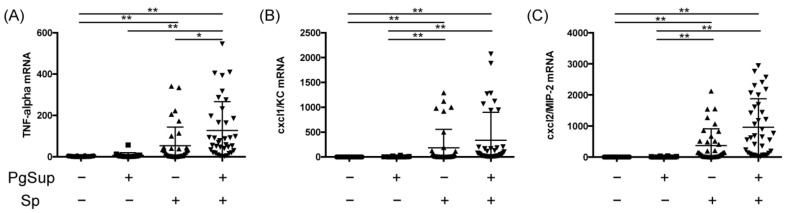
The mRNA expression of *TNF-α*, *CXCL1*, and *CXCL2* in Sp-infected mice inoculated with or without PgSup. Lung homogenates from each group were analyzed to compare (**A**) *TNF-α*, (**B**) *CXCL1*, and (**C**) *CXCL2* gene expression levels using qPCR. “+” indicates the presence of PgSup or Sp, and “−” indicates the absence of PgSup or Sp. Each group was analyzed by one-way ANOVA, and data are presented as the mean ± SD. Significant differences were analyzed using the Kruskal–Wallis test (* *p* < 0.05, ** *p* < 0.01).

To investigate the factors involved in lung inflammation, we examined the mRNA expression of inflammatory cytokines and chemokines by qPCR. The lungs of the Sp-infected group and the Sp and PgSup mixed-infected group showed significantly increased mRNA expression of *tumor necrosis factor (TNF)-α*, *CXC chemokine ligand 1 (CXCL1)*, and *CXCL2* compared with the control group (*p* < 0.01; Figure 3A–C). Whereas the PgSup group did not show increased mRNA expression of *TNF-α*, *CXCL1*, and *CXCL2* in the lungs compared with the control group, the mRNA expression of *TNF-α* in the mixed-infected group was significantly increased (2.35-fold) compared with the Sp-infected group (*p* < 0.05). The mRNA expression levels of *CXCL1* and *CXCL2* in the lungs of the mixed-infected group were increased compared with the Sp-infected group (1.82-fold and 2.59-fold, respectively; Figure 3B,C).

### 2.4. PgSup Enhanced the Production of Pro-Inflammatory Cytokines and Chemokines in the Lungs of Sp-Infected Mice

**Figure 4:**
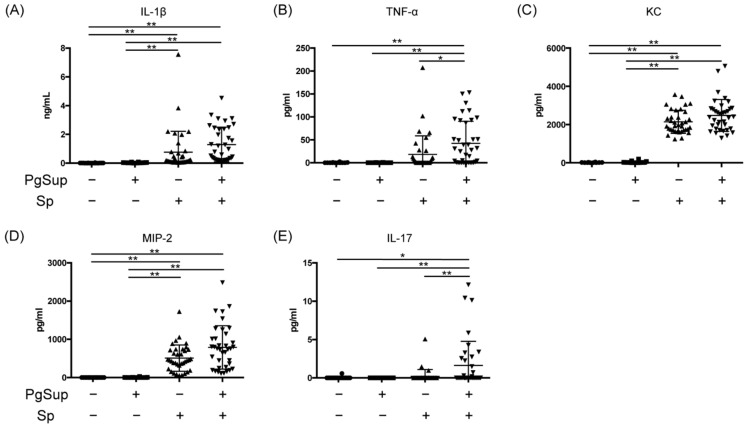
The production of IL-1β, TNF-α, KC, MIP-2, and IL-17 in Sp-infected mice inoculated with or without PgSup. The supernatants obtained by centrifugation of lung homogenates of each group were compared for (**A**) IL-1β, (**B**) TNF-α, (**C**) KC, (**D**) MIP-2, and (**E**) IL-17 production by ELISA. “+” indicates the presence of PgSup or Sp, and “−” indicates the absence of PgSup or Sp. Each point represents an individual mouse. Each group was analyzed by one-way ANOVA, and data are presented as the mean ± SD. Significant differences were analyzed using the Kruskal–Wallis test (* *p* < 0.05, ** *p* < 0.01).

We next examined the effects of PgSup on the production of inflammatory cytokines and chemokines in Sp-infected mice. The levels of IL-1β, TNF-α, keratinocyte-derived chemokines (KC), macrophage inflammatory protein–2 (MIP-2), and IL-17 in the lungs were increased in the Sp-infected and mixed-infected groups (Figure 4A–E). In the control and PgSup groups, the levels of these factors were almost undetectable. The production of TNF-α in the lungs was significantly increased in the mixed-infected group compared with the Sp-infected group (*p* < 0.05; Figure 4B). On comparing the mean values of the Sp-infected and mixed-infected groups, we found increased expression of IL-1β (1.69-fold), TNF-α (2.32-fold), KC (1.15-fold), MIP-2 (1.63-fold), and IL-17 (7.8-fold) in the mixed-infected group. Interestingly, the IL-17 production level in the mixed-infected group was significantly increased compared with the Sp-infected group (*p* < 0.01), even on Day 1 after Sp infection (Figure 4E).

## 3. Discussion

In this study, pathological examination of the lungs of mice infected with both Sp and PgSup revealed increased inflammatory cell infiltration compared with infection with Sp alone. We further showed that PgSup synergistically enhanced the gene expression and protein production of pro-inflammatory cytokines/chemokines in the lungs of Sp-infected mice. Importantly, PgSup addition alone did not cause an inflammatory reaction in the lungs, suggesting that Pg components/secretions indirectly enhance the inflammatory reaction caused by Sp infection in the lungs.

TNF-α is secreted by macrophages, natural killer cells, endothelial cells, and fibroblasts, and induces the proinflammatory cytokines IL-1β and IL-6, which are involved in innate immunity in the early stages of infection [20]. In addition, CXCL1/KC and CXCL2/MIP-2 exist in monomeric and dimeric forms and are involved in neutrophil migration by activating CXC chemokine receptor 2 (CXCR2), the CXC chemokine receptor, and binding to glycosaminoglycans [21,22]. In this study, we found that 24 h after the initiation of pneumonia (early stage of infection), PgSup synergistically increased the expression of TNF-α in the lungs of Sp-infected mice (Figure 3 and Figure 4). In addition, Sp-infected mice and Sp and PgSup mixed-infected mice showed a higher tendency to infiltrate neutrophils and macrophages into the lung compared with control and PgSup-infected mice. These suggest that the bacterial components/secretions of *P. gingivalis* promote lung inflammation of Sp-administrated mice by inducing recruitment and activation of both neutrophils and inflammatory monocytes/macrophages, which may work in concert to drive inflammation. Another important factor for neutrophils—IL-17—is produced by TH17 cells, NKT cells, neutrophils, monocytes, and NK cells, and causes neutrophil activation and migration to inflammatory sites [23,24]. The production of IL-17 in mice receiving the mixed infection was significantly increased compared with those infected with Sp alone, but the degree of IL-17 expression was low due to the early stage of infection (Figure 4). 

*P. gingivalis* plays an important role in the pathogenesis of AP, as has been reported in many clinical cases and animal models [25,26]. This bacterium expresses virulence factors such as LPS, phosphatase, fimbriae, and a cysteine protease called gingipain, which directly or indirectly activate host cells and play a major role in the formation of inflammatory foci [27,28,29,30]. In particular, there are two types of gingipains with different peptide cleavage site specificities, Arg-gingipain (Rgp) and Lys-gingipain (Kgp), which are secreted as monomers into the extracellular space and degrade extracellular matrices such as collagen (types I and IV) and fibronectin [30,31,32,33]. Rgp and Kgp exist as high molecular-weight complexes bound to hemocyte aggregates, hemoglobin-binding proteins, LPS, and phospholipids on the outer membrane, and these membrane-bound gingipain complexes exhibit more potent cytotoxic activity than monomers [28,34,35]. Gingipains also play a role in *P. gingivalis* evasion of host defense mechanisms by degrading antimicrobial peptides such as defensins, C3 and C4—involved in the complement cascade—and T-cell receptors and mediators [36]. In this study, we used bacterial culture supernatant rather than *P. gingivalis* bacteria, which are anaerobic, allowing us to investigate the synergistic effects of lung infection without being affected by the aerobic environment of the lung. Our results suggested the possibility that the lung infection in mice inoculated with *S. pneumoniae* and PgSup might be caused by gingipains produced by *P. gingivalis*, which damage the extracellular matrix of alveolar epithelial cells and facilitate the entry of *S. pneumoniae* into the tissues, thereby increasing the inflammatory reaction. Future studies are needed to investigate whether *P. gingivalis* gingipains can damage the extracellular matrix of alveolar epithelial cells, facilitating the entry of *S. pneumoniae* into the tissues and causing an increase in inflammation.

*S. pneumoniae* is known to infect the alveolar epithelium and cause pneumonia when pneumococcal surface protein A (PspA) and pneumococcal surface protein C (PspC) bind to host receptors such as the platelet-activating factor receptor (PAFR) [37]. It has also been reported that infection of airway epithelial cells with rhinoviruses enhances PAFR expression and facilitates adhesion of *S. pneumoniae* to epithelial cells [38]. Furthermore, Kamio and colleagues reported that PAFR expression was induced in human alveolar epithelial cells stimulated with PgSup, and that culture supernatant stimulation using a gingipain-deficient mutant inhibited PAFR expression and S. pneumoniae adherence to alveolar epithelial cells [39]. Similarly, in our experiment, gingipain in the PgSup may have induced the expression of PAFR and promoted the adhesion of *S. pneumoniae* to alveolar epithelium, leading to an enhanced inflammatory reaction in the lungs. Further in vitro and in vivo analyses are needed to clarify the mechanism of action of *P. gingivalis* components/secretions in pneumococcal infection.

This study has several limitations. First, to clarify the mechanism of action of *P. gingivalis* components/secretions in pneumococcal infection, further in vitro and in vivo analyses are needed. Second, since the inflammatory state changes depending on the number of *S. pneumoniae* causing the lung infection, further analyses are needed with specific numbers of infecting *S. pneumoniae*. Third, we have demonstrated that PgSup acts synergistically in the first 24 h after the initiation of pneumonia infection (early infection), but the long-term effects are not clear. Further analysis of the effects of PgSup on acquired immunity via T cells and B cells is needed using a longer-term infection model.

## 4. Materials and Methods

### 4.1. Bacterial Strains and Culture Conditions

The *S. pneumoniae* strain URF918 was provided by Professor Kazuyoshi Kawakami (Tohoku University). *S. pneumoniae* was mainly grown on blood agar plates (Trypticase Soy Agar, BD Biosciences, San Jose, CA, USA) supplemented with 5% laked sheep blood, 2.5 μg/mL hemin, and 5.0 μg/mL menadione for 24 h at 37 °C under anaerobic conditions and was then scraped and suspended in 5 mL of Trypticase Soy Broth (BD Biosciences) supplemented (sTSB) with 0.25% yeast extract, 2.5 μg/mL hemin, and 5.0 μg/mL menadione. To prepare a bacterial suspension, *S. pneumoniae* was incubated with sTSB until late logarithmic growth phase (OD_600_ = 1.0). Bacteria were then harvested by centrifugation (5000× *g*, 10 min) and resuspended in phosphate-buffered saline (PBS, pH 7.4). The OD600 of the resulting cell suspension was measured and adjusted to 1.0 by dilution with sTSB.

*P. gingivalis* ATCC 33277 was mainly grown on blood agar plates supplemented with 5% laked sheep blood, 2.5 μg/mL hemin, 5.0 μg/mL menadione, and 0.01% DTT at 37 °C under anaerobic conditions. *P. gingivalis* strains were also cultured in sTSB at 37 °C under anaerobic conditions. PgSup was obtained as previously reported [40]. Briefly, a small-scale primary culture (100 μL) of *P. gingivalis* was inoculated into fresh sTSB (5 mL) under anaerobic conditions for 24 h. The supernatant was then collected by centrifugation at 5000× *g* for 10 min at 4 °C to remove the bacteria and then were filter sterilized through a 0.22-µm pore-size membrane filter (Millex GV, Merck Millipore Ltd., Carrigtwohill, Co., Cork, Ireland).

### 4.2. Mice

C57BL/6J mice were purchased from Japan SLC Inc. (Shizuoka, Japan) at 6 weeks of age. All mice were maintained under specific-pathogen-free conditions and were used between 6 and 8 weeks of age. The experiments were approved by the Aichi Gakuin University Institutional Animal Care and Use Committee and performed according to institutional guidelines.

### 4.3. Design of Animal Experiments

To reproduce AP, mice were anesthetized with mixed anesthetic agents consisting of medetomidine hydrochloride, midazolam, and butorphanol tartrate (injected intraperitoneally at 0.1 mL per 10 g of mouse body weight). After the throat was incised, mice were administered a bacterial suspension directly through the incised bronchus.

In mixed-infection experiments with Sp and PgSup, 0.05 mL of the bacterial suspension was inoculated into each mouse. 

*S. pneumoniae* was mixed with equal amounts of PgSup or sTSB before inoculation into mice. The final concentration of *S. pneumoniae* administered to mice was 4.4 × 10^6^–7.0 × 10^7^ CFU/mL (2.2 × 10^5^–3.5 × 10^6^ CFU/mouse). Mice were euthanized 24 h post-infection, and the lungs were collected. Mice were treated with sTSB (*n* = 18), PgSup (*n* = 18), Sp (*n* = 36), or mixed administration (*n* = 37).

### 4.4. Histological Analysis

Histological sections of the left lung were prepared 24 h after intratracheal administration of Sp and PgSup to mice. The lung samples were fixed in 10% formalin solution for 24 h. Samples were rinsed in running tap water for 30 min, followed by dehydration and paraffin embedding. The samples were cut using a microtome (REM-700, YAMATO KOHKI, Saitama, Japan) to a thickness of 5 µm and placed on glass slides (APS-05, Matsunami Glass, Osaka, Japan). HE staining was performed in accordance with routine protocols. The sections were stained with hematoxylin solution for 5 min after deparaffinization and hydration and then washed in distilled water. Then, the sections were stained with eosin solution for 1 min, dehydrated using graded alcohol, and cleaned by xylene. The slide glasses were examined under a microscope (BX51, Olympus, Tokyo, Japan) and photographed using a digital camera (DP70, Olympus). Image analysis was performed using ImageJ to determine the percentage of interstitium in areas of HE-stained histological sections (×40). HE-stained pathology of lung tissue collected from two infection experiments (*n* = 12 per group) was used for analysis.

### 4.5. Cell Preparation and Flow Cytometric Analysis

The lung was perfused with PBS through the right ventricle before excision from the mice. The excised lung tissue, separated from all the associated lymph nodes, was minced by a gentleMACS™ dissociator (Miltenyi Biotec, Bergisch Gladbach, Germany) and incubated for 1 h at 37 °C in 5 mL of PBS containing 1.0% FBS, 125 U/mL of collagenase I (Sigma, St. Louis, MO, USA), 60 U/mL of DNase I (Sigma), and 60 U/mL of hyaluronidase (Sigma). Single-cell suspensions were prepared by passing through a 30 µm stainless steel mesh. The lung homogenized cells were pretreated with culture supernatant from the 2.4G2 hybridoma-producing mAb against FcγR II/III (Fc blocker) and then were surface stained with FITC-conjugated anti-Gr-1 and PE-conjugated anti-CD11b mAbs (BD Biosciences) to detect macrophages and neutrophils. Cells were analyzed with a FACSCanto flow cytometer with DIVA software (BD Biosciences). 

### 4.6. Bacterial Numbers in the Lung

After the mice had been euthanized, the right lungs were collected from the Sp-only-infected and Sp and PgSup mixed-infected mice at Day 1 after infection. Then, 0.1 mL of homogenized right lung tissue was plated onto blood agar plates at a limiting dilution, and the CFU per gram of lung tissue was measured.

### 4.7. RNA Extraction and Quantitative Polymerase Chain Reaction (qPCR) Analysis

The right lung tissue collected after euthanization for the Sp, PgSup-infected, and control mice was transferred to MACS M Tubes (Miltenyi Biotec) containing 2 mL of PBS and homogenized using a gentleMACS™ dissociator (Miltenyi Biotec). After homogenization, centrifugation was performed at 320× *g* for 5 min at 4 °C; the Nucleospin RNA kit (Macherey-Nagel Inc., Bethlehem, PA, USA) was used according to the manufacturer’s instructions, and the resulting pellets were used to extract total RNA. The NanoDrop Lite (Thermo Fisher Scientific, Wilmington, DE, USA) was then used to assess the purity and concentration by calculating the A230/A260 and A260/A280 ratios, respectively. The cDNA was synthesized from RNA by the action of RiverTraAce^®^ (Toyobo Co., Ltd., Osaka, Japan). To quantify mRNA, quantitative PCR was performed using the TaqMan gene expression assay (Thermo Fisher Scientific) for mouse *Tnf-α* (Mm00443258_m1), *Cxcl1* (Mm04207460_m1), *Cxcl2* (Mm00436450_m1), and *18S rRNA* (Hs99999901_s1) with the TaqMan Universal PCR Master Mix (Thermo Fisher Scientific). The cDNA was amplified and quantified using the StepOnePlus™ real-time PCR system. The optimal reaction conditions for the one-step assay were as follows: a denaturation step at 95 °C for 10 min, followed by 40 cycles at 95 °C for 15 s and 60 °C for 40 s. Expression data were normalized to the geometric mean of the housekeeping gene, *18S rRNA*, to control the variability in expression levels and were analyzed using the 2^−^^ΔΔCT^ method.

### 4.8. Enzyme-Linked Immunosorbent Assay (ELISA)

The right lungs recovered from infected mice were homogenized with the gentleMACS™ Dissociator and then centrifuged (320× *g* for 5 min at 4 °C), and the supernatant was recovered to detect the levels of IL-1β, TNF-α, KC, MIP-2, and IL-17. Quantikine mouse IL-1β, TNF-α, KC, MIP-2, and IL-17 ELISA kits (R&D Systems, Minneapolis, MN, USA) were used according to the manufacturer’s instructions. The minimum detectable concentration was 1–5 pg/mL.

### 4.9. Statistical Analysis

Data were analyzed using PASW Statistics software (version 18.0; SPSS Japan, Tokyo, Japan). Differences among groups were examined by one-way analysis of variance (ANOVA) and the Kruskal–Wallis test. Comparisons of two independent groups were performed using unpaired *t*-tests. Data are expressed as the mean ± standard deviation (SD). Significance was accepted at *p* < 0.05.

## 5. Conclusions

This study demonstrated that PgSup enhances pneumonia caused by *S. pneumoniae*. This suggests the pathogenic factors derived from *P. gingivalis* may play an important role in the effects of periodontitis on exacerbation of respiratory tract infections such as AP. Clarification of these phenomena and the mechanisms involved is expected to provide evidence that treatments targeted at oral periodontopathic bacteria will inhibit the development of AP. Further studies are needed to elucidate the detailed mechanisms of interactions between *P. gingivalis* and *S. pneumoniae*.

## Figures and Tables

**Table 1:** The absolute number and frequency of neutrophils and macrophages in the lung after administration. Mice were administrated with sTSB, PgSup, Sp, or Sp-PgSup mixed. After 24 h, the whole cells of the lungs were counted by microscopy and then the number of the lung infiltrating cells calculated after being analyzed by flow cytometry. The same superscript within the column indicates that there is a significant difference among treatments (*p* < 0.05, *t*-test).

Group	Whole Cells (Cells)	Neutrophils (Cells)	Macrophages (Cells)	Neutrophils (%)	Macrophages (%)
Control	6.15 ± 0.78 × 10^6^	3.51 ± 1.07 × 10^5 b^	0.85 ± 0.04 × 10^6 d^	5.62 ± 1.16	14.06 ± 1.71
PgSup	5.90 ± 0.67 × 10^6 a^	3.99 ± 1.65 × 10^5 c^	0.90 ± 0.08 ×10^6 e,f^	6.67 ± 2.14	15.31 ± 1.05
Sp	7.98 ± 1.11 × 10^6 a^	5.67 ± 2.67 × 10^5^	1.52 ± 0.29 × 10^6 e^	7.38 ± 3.94	18.95 ± 1.99
Sp + PgSup	7.78 ± 1.61 × 10^6^	8.09 ± 1.65 × 10^5 b,c^	1.32 ± 0.17 × 10^6 d,f^	10.41 ± 0.58	17.19 ± 1.58

## Data Availability

Data are available on request.
